# Supplementary material for: Association of liver fibrosis biomarkers with overall and CVD mortality in the Korean population: The Dong-gu study
Source: PLoS One. 2022 Dec 13;17(12):e0277729. doi: 10.1371/journal.pone.0277729 (PMC9747044; doi:10.1371/journal.pone.0277729)
Supplement: Attachment — Submitted filename: Response to Reviewers.docx [file pone.0277729.s001.docx]

Journal Requirements:

When submitting your revision, we need you to address these additional requirements.

1. Please ensure that your manuscript meets PLOS ONE's style requirements, including those for file naming. The PLOS ONE style templates can be found at 
https://journals.plos.org/plosone/s/file?id=wjVg/PLOSOne_formatting_sample_main_body.pdf and 
https://journals.plos.org/plosone/s/file?id=ba62/PLOSOne_formatting_sample_title_authors_affiliations.pdf

Additional Editor Comments:
* Please format the title page, manuscript, and references according to PLOS ONE guidelines.

Answer: According to PLOS ONE guidelines, we revised the title page, manuscript, and references

* In tables, when expressing the confidence interval, please use en dash. The tilde is not universally used for expressing the range. Also please correct other type errors.

Answer: We revised the tilde into en dash in tables.

Table 2. Characteristics of the subjects according to the level of NFS, FIB-4, APRI, and BARD score

| Variable | NFS | | | FIB-4 | | | APRI | | | BARD score | | |
| --- | --- | --- | --- | --- | --- | --- | --- | --- | --- | --- | --- | --- |
|  | Low risk  (<-1.455) | Intermediate risk  (-1.455-0.676) | High risk  (>0.676) | Low risk  (<1.30) | Intermediate risk  (1.30-2.67) | High risk  (>2.67) | Low risk  (<0.5) | Intermediate risk  (0.5-1.5) | High risk  (>1.5) | Low risk  (0-1) | Intermediate risk  (2-3) | High risk  (4) |
| N (%) | 4,444 (57.7) | 3,058 (39.7) | 200 (2.6) | 3,175 (41.2) | 4,090 (53.1) | 437 (5.7) | 7,245 (94.1) | 420 (5.5) | 37 (0.5) | 489 (6.3) | 7,017 (91.1) | 196 (2.5) |
| Follow-up duration (years) | 10.6±1.9 | 9.9±2.4 | 8.2±3.3^*^ | 10.6±1.9 | 10.1±2.2 | 8.9±3.1^*^ | 10.3±2.2 | 9.8±2.6 | 8.0±3.6^*^ | 10.9±2.2 | 10.2±2.2 | 10.3±1.8^*^ |
| Male (%) | 1,225 (27.6) | 1,280 (41.9) | 90 (45.0)^*^ | 812 (25.6) | 1,561 (38.2) | 222 (50.8)^*^ | 2,401 (33.1) | 178 (42.4) | 16 (43.2)^*^ | 224 (45.8) | 2,327 (33.2) | 44 (22.4)^*^ |
| Age (years) | 62.8±7.5 | 68.6±7.7 | 73.3±8.0^*^ | 61.7±7.2 | 67.5±7.7 | 72.2±8.1^*^ | 65.3±8.2 | 66.8±8.4 | 67.5±6.9^*^ | 63.0±7.2 | 65.5±8.3 | 66.6±7.8^*^ |
| Height (cm) | 157.0±7.8 | 157.9±8.5 | 157.1±10.0^*^ | 156.8±7.7 | 157.7±8.4 | 158.5±9.3^*^ | 157.3±8.2 | 158.7±8.3 | 158.2±9.9^**^ | 159.9±8.0 | 157.3±8.2 | 155.3±7.9^*^ |
| Weight (kg) | 59.3±8.6 | 62.0±9.5 | 62.4±10.7^*^ | 60.6±8.7 | 60.4±9.3 | 60.0±10.0 | 60.3±9.1 | 62.9±9.8 | 61.2±12.3^*^ | 64.2±9.3 | 59.9±8.9 | 72.0±8.2^*^ |
| BMI (kg/m^2^) | 24.0±2.7 | 24.8±3.1 | 25.2±3.3^*^ | 24.6±2.8 | 24.3±3.0 | 23.8±3.2^*^ | 24.3±2.9 | 24.9±3.4 | 24.3±3.2^*^ | 25.1±2.6 | 24.2±2.8 | 29.8±1.7^*^ |
| Waist circumference (cm) | 87.0±8.3 | 89.2±8.9 | 90.2±9.0^*^ | 88.5±8.2 | 87.7±8.8 | 86.9±9.7^*^ | 87.9±8.5 | 89.5±9.7 | 87.8±9.4^**^ | 90.9±7.3 | 87.4±8.5 | 100.1±6.4^*^ |
| Smoking (%) | 372 (8.4) | 261 (8.5) | 16 (8.0)^*^ | 280 (8.8) | 335 (8.2) | 34 (7.8)^*^ | 606 (8.4) | 38 (9.0) | 5 (13.5) | 53 (10.8) | 577 (8.2) | 19 (9.7)^*^ |
| Alcohol intake (%) | 2,292 (51.6) | 1,537 (50.3) | 115 (57.5)^**^ | 1,600 (50.4) | 2,112 (51.6) | 232 (53.1) | 3,716 (51.3) | 207 (49.3) | 21 (56.8) | 210 (42.9) | 3,620 (51.6) | 114 (58.2)^**^ |
| ^a^Regular walking (%) | 2,806 (63.1) | 1,885 (61.6) | 118 (59.0) | 1,950 (61.4) | 2,593 (63.4) | 266 (60.9) | 4,516 (62.3) | 269 (64.0) | 24 (64.9) | 312 (63.8) | 4,393 (62.6) | 104 (53.1)^**^ |
| ^b^Hypertension (%) | 1,722 (38.8) | 1,555 (50.9) | 123 (61.5)^*^ | 1,287 (40.5) | 1,882 (46.0) | 231 (52.9)^*^ | 3,154 (43.5) | 225 (53.6) | 21 (56.8)^*^ | 231 (47.2) | 3,032 (43.2) | 137 (69.9)^*^ |
| ^c^Diabetes (%) | 389 (8.8) | 1,095 (35.8) | 125 (62.5)^*^ | 699 (22.0) | 815 (19.9) | 95 (21.7) | 1,478 (20.4) | 115 (27.4) | 16 (43.2)^*^ | 185 (37.8) | 1,228 (17.5) | 196 (100.0)^*^ |
| Systolic BP (mm Hg) | 121.6±16.7 | 124.6±16.7 | 126.6±18.1^*^ | 121.6±16.6 | 123.6±16.9 | 125.8±17.0^*^ | 122.8±16.8 | 124.3±16.9 | 125.1±15.8 | 123.0±14.7 | 122.8±17.0 | 126.7±16.4^**^ |
| HbA_1c_ (%) | 5.7±0.8 | 6.1±1.1 | 6.2±1.1^*^ | 5.9±1.0 | 5.8±0.9 | 5.7±0.8^*^ | 5.8±0.9 | 5.9±1.0 | 5.7±1.0 | 6.3±1.4 | 5.8±0.9 | 7.0±1.1^*^ |
| Total cholesterol (mg/dL) | 207.5±38.7 | 196.7±40.3 | 190.0±43.7^*^ | 206.6±40.1 | 201.0±39.2 | 190.7±41.5^*^ | 203.2±39.5 | 197.1±44.6 | 181.3±48.5^*^ | 202.9±44.6 | 202.9±39.5 | 197.1±42.8 |
| Triglycerides (mg/dL) | 143.9±92.0 | 134.4±80.9 | 125.2±81.6^*^ | 149.7±93.1 | 134.0±83.5 | 119.3±74.7^*^ | 139.1±85.4 | 149.9±118.2 | 118.2±95.4^**^ | 167.1±107.7 | 136.9±85.4 | 168.6±93.0^*^ |
| HDL cholesterol (mg/dL) | 51.8±11.7 | 50.4±11.7 | 49.5±12.9^*^ | 50.9±11.4 | 51.4±11.8 | 51.5±12.4 | 51.2±11.6 | 51.0±13.4 | 51.9±16.5 | 49.8±11.7 | 51.4±11.7 | 47.9±10.1^*^ |
| AST (IU/L) | 23.1±13.0 | 24.8±16.7 | 33.8±32.7^*^ | 20.9±5.2 | 24.6±7.6 | 41.8±55.6^*^ | 22.4±5.4 | 41.4±15.2 | 146.6±151.9^*^ | 26.3±14.8 | 23.8±15.5 | 28.4±14.3^*^ |
| ALT (IU/L) | 20.7±13.1 | 19.2±14.8 | 17.1±16.3^*^ | 20.2±9.6 | 19.2±10.9 | 26.2±39.7^*^ | 18.6±8.1 | 37.8±23.9 | 101.9±103.2^*^ | 39.1±23.3 | 18.5±11.8 | 25.0±15.2^*^ |
| GGT (IU/L) | 27.8±33.0 | 29.4±34.9 | 71.3±226.8^*^ | 27.4±32.4 | 28.1±28.7 | 58.9±166.4^*^ | 26.9±28.5 | 54.3±62.6 | 276.1±503.0^*^ | 44.2±65.7 | 28.3±48.9 | 37.6±26.9^*^ |
| Platelet (IU/L) | 278.5±57.9 | 217.6±44.9 | 169.3±56.2^*^ | 291.1±58.4 | 230.1±43.7 | 164.2±46.6^*^ | 256.2±59.5 | 180.5±51.8 | 132.0±72.7^*^ | 246.1±60.3 | 251.8±62.1 | 255.6±69.3^*^ |
| Albumin (IU/L) | 4.5±0.2 | 4.4±0.3 | 4.3±0.4^*^ | 4.5±0.3 | 4.5±0.3 | 4.4±0.4^*^ | 4.5±0.3 | 4.5±0.3 | 4.0±0.6^*^ | 4.6±0.3 | 4.5±0.3 | 4.4±0.2^*^ |
| NFS | -2.4±0.7 | -0.7±0.5 | 1.4±0.8^*^ | -2.5±0.9 | -1.2±0.8 | 0.4±1.0^*^ | -1.7±1.1 | -0.5±1.2 | 0.9±1.8^*^ | -2.1±1.0 | -1.6±1.1 | -0.3±1.2^*^ |
| FIB-4 | 1.2±0.3 | 1.9±0.6 | 4.4±4.2^*^ | 1.0±0.2 | 1.7±0.3 | 3.9±2.9^*^ | 1.4±0.5 | 2.8±1.2 | 9.8±7.4^*^ | 1.1±0.5 | 1.6±1.0 | 1.8±2.2^*^ |
| APRI | 0.3±0.2 | 0.3±0.2 | 0.8±1.0^*^ | 0.2±0.1 | 0.3±0.1 | 0.8±1.0^*^ | 0.3±0.1 | 0.7±0.2 | 3.2±2.1^*^ | 0.3±0.3 | 0.3±0.3 | 0.4±0.5^*^ |
| BARD score | 2.0±0.6 | 2.4±0.7 | 2.9±0.7^*^ | 2.1±0.8 | 2.2±0.6 | 2.3±0.6^*^ | 2.2±0.7 | 2.2±0.9 | 2.4±0.9 | 0.5±0.5 | 2.2±0.4 | 4.0±0.0^*^ |
| Deceased by all-cause (%) | 434 (9.8) | 626 (20.5) | 81 (40.5)^*^ | 307 (9.7) | 689 (16.8) | 145 (33.2)^*^ | 1,033 (14.3) | 91 (21.7) | 17 (45.9)^*^ | 62 (12.7) | 1,052 (15.0) | 27 (13.8) |
| Deceased by CVD (%) | 13 (0.3) | 34 (1.1) | 4 (2.0)^*^ | 11 (0.3) | 32 (0.8) | 8 (1.8)^*^ | 50 (0.7) | 1 (0.2) | 0 (0.0) | 4 (0.8) | 47 (0.7) | 0 (0.0) |

All values are given as *n* (%) or mean ± standard deviation.

^*^p<0.001, ^**^p<0.05

NFS, NAFLD fibrosis score; BMI, body mass index; BP, blood pressure; HbA_1c_, glycated hemoglobin; HDL, high-density lipoprotein; AST, aspartate aminotransferase; ALT, alanine aminotransferase; GGT, gamma-glutamyl transpeptidase; FIB-4, Fibrosis-4 score; APRI, aspartate aminotransferase to platelet ratio index

^a^Subjects who performed walking for more than 30 minutes at one time and more than 5 times per week

^b^Hypertension was defined as systolic blood pressure ≥140mm Hg or diastolic blood pressure ≥90mm Hg or taking anti-hypertension medication.

^c^Diabetes was defined as fasting serum glucose ≥126 mg/dl or taking insulin or oral diabetes medication.

Table 3. Hazard ratios for overall and CVD mortality according to the level of NFS, FIB-4, APRI, and BARD score

| Variables | | NFS | | FIB-4 | | APRI | | BARD score | |
| --- | --- | --- | --- | --- | --- | --- | --- | --- | --- |
|  |  | Non-adjusted | Adjusted^a^ | Non-adjusted | Adjusted^b^ | Non-adjusted | Adjusted^c^ | Non-adjusted | Adjusted^d^ |
| Overall mortality | Low risk | Reference | Reference | Reference | Reference | Reference | Reference | Reference | Reference |
|  | Intermediate risk | 2.3 (2.0–2.6) | 1.9 (1.7–2.2) | 1.9 (1.6–2.1) | 1.7 (1.5–2.0) | 1.6 (1.3–2.0) | 1.3 (1.0–1.6) | 1.3 (1.0–1.7) | 1.2 (0.9–1.6) |
|  | High risk | 5.8 (4.5–7.3) | 4.3 (3.3–5.5) | 4.3 (3.6–5.3) | 3.5 (2.9–4.4) | 4.4 (2.7–7.1) | 3.5 (2.1–5.8) | 1.2 (0.8–1.9) | 0.9 (0.6–1.4) |
| CVD mortality | Low risk | Reference | Reference | Reference | Reference | Reference | Reference | Reference | Reference |
|  | Intermediate & high risk | 4.3 (2.3–8.2) | 3.6 (1.8–6.9) | 2.7 (1.4–5.3) | 2.6 (1.3–5.2) | 0.3 (0.5–2.5) | 0.3 (0.1–1.9) | 1.2 (0.4–3.3) | 0.5 (0.1–1.5) |

NFS, NAFLD fibrosis score; FIB-4, Fibrosis-4 score; APRI, aspartate aminotransferase to platelet ratio index

^a^adjusted by sex, log-waist circumference, smoking, alcohol intake, regular walking, hypertension, log-SBP, log-HbA_1c_, log-total cholesterol, log-triglyceride, log-HDL, and log-GGT;

^b^adjusted by sex, log-waist circumference, smoking, alcohol intake, regular walking, hypertension, diabetes, log-SBP, log-HbA_1c_, log-total cholesterol, log-triglyceride, log-HDL, and log-GGT;

^c^adjusted by sex, age, log-waist circumference, smoking, alcohol intake, regular walking, hypertension, diabetes, log-SBP, log-HbA_1c_, log-total cholesterol, log-triglyceride, log-HDL, and log-GGT;

^d^adjusted by sex, age, log-waist circumference, smoking, alcohol intake, regular walking, hypertension, log-SBP, log-HbA_1c_, log-total cholesterol, log-triglyceride, log-HDL, and log-GGT;

* It would be more informative to draw Kaplan-Meier curve to compare the probability of death between low, intermediate, and high risk.

Answer: According to your suggestion, we added Kaplan-Meier survival estimates like below.

In Kaplan-Meier survival estimates (Fig 1), overall survival was lower at higher risk for NFS, FIB-4, and APRI (Log-rank test p<0.001), but not for BARD scores (Log-rank test p=0.126).

**Fig 1. Kaplan-Meier survival estimates between NFS, FIB-4, APRI, and BARD score for overall mortality**

* Ascertainment of death-"Linking with National Statistical Office registries yielded death information. Until December". What this sentence means? Only death event could be confirmed? Not date?

Answer: There was an error in the description. We revised the sentence like below

Linking with National Statistical Office registries yielded death information. The date of death was ascertained until December 31, 2020.

* ICD-10 I20-25 cover cardiovascular disease. But, I60-69 cover "cerebrovascular disease". Although they share same risk factors, cardiovascular disease is a disease of the blood vessels in the heart, and cerebrovascular disease is a disease of the blood vessels in the brain. Therefore, it seems irresolute to combine them into cardiovascular disease. * Moreover, I wonder the accuracy of the disease-specific death. For example, ICD-10 I20 is angina pectoris which could be not severe and I25 is a kind of chronic disease. Then, how the authors could be confident on their definition on cardiovascular death? Isn't there another possible main cause of death such as malignancy?

Answer: We agree with the editor. However, there is no definitive ICD-10 classification for CVD and each study or institution defines it slightly differently. In the US CDC study, diseases of heart (ICD–10 codes I00–I09, I11, I13, I20–I51), essential hypertension and hypertensive renal disease (I10, I12, I15) and cerebrovascular diseases (I60–169) were all classified as CVD (Miniño, A., and R. Klein. Mortality From Major Cardiovascular Diseases: United States, 2007. *National Center for Health Statistics* (2010): 2020-07.). Another study defines death from CVD as death from either heart disease or cerebrovascular disease (ICD-10 codes I00-I69) classified as CVD (Rong S, Snetselaar L, Xu G, et al. Association of Skipping Breakfast With Cardiovascular and All-Cause Mortality. *J Am Coll Cardiol*. 2019 Apr, 73 (16) 2025–2032. https://doi.org/10.1016/j.jacc.2019.01.065). The other study classified ICD-10 code I20-I25 as CVD (Shah, Nilay S., et al. Cardiovascular and Cerebrovascular Disease Mortality in Asian American Subgroups. *Circulation: Cardiovascular Quality and Outcomes* 15.5 (2022): e008651).

Based on your suggestion, we have modified the classification of CVD to ICD-10 code I20–25. Due to the change in the classification of CVD, the number of CVD deaths was low, so we merged the intermediate and high risk groups in calculation ORs of CVD mortality in Table 3.

Table 3. Hazard ratios for overall and CVD mortality according to the level of NFS, FIB-4, APRI, and BARD score

| Variables | | NFS | | FIB-4 | | APRI | | BARD score | |
| --- | --- | --- | --- | --- | --- | --- | --- | --- | --- |
|  |  | Non-adjusted | Adjusted^a^ | Non-adjusted | Adjusted^b^ | Non-adjusted | Adjusted^c^ | Non-adjusted | Adjusted^d^ |
| Overall mortality | Low risk | Reference | Reference | Reference | Reference | Reference | Reference | Reference | Reference |
|  | Intermediate risk | 2.3 (2.0–2.6) | 1.9 (1.7–2.2) | 1.9 (1.6–2.1) | 1.7 (1.5–2.0) | 1.6 (1.3–2.0) | 1.3 (1.0–1.6) | 1.3 (1.0–1.7) | 1.2 (0.9–1.6) |
|  | High risk | 5.8 (4.5–7.3) | 4.3 (3.3–5.5) | 4.3 (3.6–5.3) | 3.5 (2.9–4.4) | 4.4 (2.7–7.1) | 3.5 (2.1–5.8) | 1.2 (0.8–1.9) | 0.9 (0.6–1.4) |
| CVD mortality | Low risk | Reference | Reference | Reference | Reference | Reference | Reference | Reference | Reference |
|  | Intermediate & high risk | 4.3 (2.3–8.2) | 3.6 (1.8–6.9) | 2.7 (1.4–5.3) | 2.6 (1.3–5.2) | 0.3 (0.5–2.5) | 0.3 (0.1–1.9) | 1.2 (0.4–3.3) | 0.5 (0.1–1.5) |

NFS, NAFLD fibrosis score; FIB-4, Fibrosis-4 score; APRI, aspartate aminotransferase to platelet ratio index

^a^adjusted by sex, log-waist circumference, smoking, alcohol intake, regular walking, hypertension, log-SBP, log-HbA_1c_, log-total cholesterol, log-triglyceride, log-HDL, and log-GGT;

^b^adjusted by sex, log-waist circumference, smoking, alcohol intake, regular walking, hypertension, diabetes, log-SBP, log-HbA_1c_, log-total cholesterol, log-triglyceride, log-HDL, and log-GGT;

^c^adjusted by sex, age, log-waist circumference, smoking, alcohol intake, regular walking, hypertension, diabetes, log-SBP, log-HbA_1c_, log-total cholesterol, log-triglyceride, log-HDL, and log-GGT;

^d^adjusted by sex, age, log-waist circumference, smoking, alcohol intake, regular walking, hypertension, log-SBP, log-HbA_1c_, log-total cholesterol, log-triglyceride, log-HDL, and log-GGT;

* Please discuss the possible mechanisms and experimental studies around the negative effect of liver fibrosis on the clinical outcomes.

Answer: According to your suggestion, we discuss like below.

Liver fibrosis is developed by a variety of etiologies, such as hepatitis B and C virus infections, metabolic disorders, and excessive alcohol intake [25]. Fibrosis is a long-term, staggering wound healing process that results increased deposition of extracellular matrix [26]. Persistent fibrosis causes cirrhosis and stiffness of the liver, impairing its physiological function, which can eventually lead to cirrhosis of the liver, end-stage liver disease, or hepatocellular cancer [25, 27]. In a multicenter cohort study of 320 NAFLD patients, higher liver fibrosis is associated with increased risk for liver related complications such as gastroesophageal bleeding, hepatopulmonary syndrome, cirrhosis complication, hepatocellular carcinoma, and liver transplantation [9]. Moreover, other epidemiologic studies have indicated that liver fibrosis is associated with aortic stiffness [28], heart failure, atrial fibrillation, and coronary heart disease [29-32].

* Several additional limitations have been raised by Reviewers. Please separate the paragraph to individually discuss the study limitations. And, please be more specific and clear, not to be ambiguous on this section.

Answer: According to your suggestion, we revised like below.

This is a well-established, large general population cohort study with an extremely high follow-up rate. Furthermore, to the best of our knowledge, this is the first study to compare the performance of liver fibrosis biomarkers in a Korean population. However, this study has some limitations. First, accurate information about liver fibrosis was lacking due to the general population's lack of access to liver biopsy. Second, data on the severity of hypertension and diabetes were not available. Instead, SBP and HbA1c were used as surrogates to adjust for blood pressure and diabetes severity. Third, since all potential confounding factors, such as progression and reversal of liver fibrosis, and changes in body mass index (BMI) and NAFLD, were not adjusted for, residual confounding may affect the main results. Finally, information on drugs for hypertension, diabetes, and hyperlipidemia has not been measured. Recently, some studies reported that the use of statin[40], angiotensin converting enzyme inhibitor (ACEI)[41], and diabetic medication[42] reduce the risk of advanced fibrosis.

Reviewer #1: This Is a well-written study based on a large population sample with a long follow-up showing that liver fibrosis biomarkers are associated to overall and cardiovascular mortality.
Results from this study confirm notions from previous studies showing that non invasive liver fibrosis indexes were correlated with cardiovascular risk and events (e.g. Pisetta, C.; Chillè, C.; Pelizzari, G.; Pigozzi, M.G.; Salvetti, M.; Paini, A.; Muiesan, M.L.; De Ciuceis, C.; Ricci, C.; Rizzoni, D. Evaluation of Cardiovascular Risk in Patient with Primary Non-alcoholic Fatty Liver Disease. High Blood Press. Cardiovasc. Prev. 2020, 27, 321–330.
Ballestri S, Mantovani A, Baldelli E, Lugari S, Maurantonio M, Nascimbeni F, Marrazzo A, Romagnoli D, Targher G, Lonardo A. Liver Fibrosis Biomarkers Accurately Exclude Advanced Fibrosis and Are Associated with Higher Cardiovascular Risk Scores in Patients with NAFLD or Viral Chronic Liver Disease. Diagnostics (Basel). 2021 Jan 9;11(1):98. doi: 10.3390/diagnostics11010098. PMID: 33435415; PMCID: PMC7827076.
Baratta, F.; Pastori, D.; Angelico, F.; Balla, A.; Paganini, A.M.; Cocomello, N.; Ferro, D.; Violi, F.; Sanyal, A.J.; Del Ben, M. Nonalcoholic Fatty Liver Disease and Fibrosis Associated With Increased Risk of Cardiovascular Events in a Prospective Study. Clin. Gastroenterol. Hepatol. 2020, 18, 2324–2331.e4).
NAFLD is an epidemic thrombofilic condition thus many patients with advance liver disease will suffer from cardiovascular disease and will be candidate to treatment (Spinosa, M.; Stine, J.G. Nonalcoholic Fatty Liver Disease-Evidence for a Thrombophilic State? Curr. Pharm. Des. 2020, 26, 1036–1044. Ballestri, S.; Capitelli, M.; Fontana, M.C.; Arioli, D.; Romagnoli, E.; Graziosi, C.; Lonardo, A.; Marietta, M.; Dentali, F.; Cioni, G. Direct Oral Anticoagulants in Patients with Liver Disease in the Era of Non-Alcoholic Fatty Liver Disease Global Epidemic: A Narrative Review. Adv. Ther. 2020, 37, 1910–1932.).
Please discuss and add appropriate literature references.

Answer: According to your suggestion, we added references in discussion like below.

Liver fibrosis is developed by a variety of etiologies, such as hepatitis B and C virus infections, metabolic disorders, and excessive alcohol intake [25]. Fibrosis is a long-term, staggering wound healing process that results increased deposition of extracellular matrix [26]. Persistent fibrosis causes cirrhosis and stiffness of the liver, impairing its physiological function, which can eventually lead to cirrhosis of the liver, end-stage liver disease, or hepatocellular cancer [25, 27]. In a multicenter cohort study of 320 NAFLD patients, higher liver fibrosis is associated with increased risk for liver related complications such as gastroesophageal bleeding, hepatopulmonary syndrome, cirrhosis complication, hepatocellular carcinoma, and liver transplantation [9]. Moreover, other epidemiologic studies have indicated that liver fibrosis is associated with aortic stiffness [28], heart failure, atrial fibrillation, and coronary heart disease [29-32].

30.	Baratta F, Pastori D, Angelico F, Balla A, Paganini AM, Cocomello N, et al. Nonalcoholic fatty liver disease and fibrosis associated with increased risk of cardiovascular events in a prospective study. Clin Gastroenterol Hepatol. 2020;18:2324-2331.e4. https://doi.org/10.1016/j.cgh.2019.12.026.

31.	Pisetta C, Chillè C, Pelizzari G, Pigozzi MG, Salvetti M, Paini A, Muiesan ML, et al. Evaluation of cardiovascular risk in patient with primary non-alcoholic fatty liver disease. High Blood Press Cardiovasc Prev 2020;27:321-330. https://doi.org/10.1007/s40292-020-00389-8.

32.	Ballestri S, Mantovani A, Baldelli E, Lugari S, Maurantonio M, Nascimbeni F, et al. Liver fibrosis biomarkers accurately exclude advanced fibrosis and are associated with higher cardiovascular risk scores in patients with NAFLD or viral chronic liver disease. Diagnostics 2021;11:98. https://doi.org/10.3390/diagnostics11010098.

Reviewer #2: This study aimed to assess associations of NFS, FIB-4, APRI and BARD scores with mortality using a large cohorts 7,702 subjects. The main results were that NFS, FIB-4, and APRI were significantly associated with overall and CVD mortality with median duration of follow-up 10.3 years.
Basically, it is quite difficult to evaluate associations of noninvasive fibrosis markers with mortality in the longitudinal cohort because lots of potential, but important confounders cannot be controlled such as change of BMI, NAFLD, even fibrosis reversal and progression during time course of about 10 years in this study. Furthermore, regarding CVD mortality, exposure to statin use and control of diabetes (new onset during periods) were not considered in the Cox analyses.

Answer: According to your suggestion, we revised like below.

However, this study has some limitations. First, accurate information about liver fibrosis was lacking due to the general population's lack of access to liver biopsy. Second, data on the severity of hypertension and diabetes were not available. Instead, SBP and HbA_1c_ were used as surrogates to adjust for blood pressure and diabetes severity. Third, since all potential confounding factors, such as progression and reversal of liver fibrosis, and changes in body mass index (BMI) and NAFLD, were not adjusted for, residual confounding may affect the main results. Finally, information on drugs for hypertension, diabetes, and hyperlipidemia has not been measured. Recently, some studies reported that the use of statin[40], angiotensin converting enzyme inhibitor (ACEI)[41], and diabetic medication[42] reduce the risk of advanced fibrosis.

Reviewer #3: Seong-Woo Choi et al evaluated the associations of liver fibrosis biomarkers [non-alcoholic fatty liver disease fibrosis score (NFS), fibrosis-4 (FIB-4), aspartate aminotransferase/platelet ratio index (APRI),
and BARD score] with mortality in Korean adults aged ≥50 years. Furthermore, the Harrell's c-index revealed that the NFS and FIB-4 were more predictive of overall and CVD mortality than the APRI and BARD score. The NFS, FIB-4, and APRI were significantly associated with overall and CVD mortality. This is a very interesting study with a long follow-up period and a large sample size. Although the relationship between NAFLD and CVD is certain. NAFLD usually has high BMI, abnormal lipid metabolism, hypertension, etc., the scientific value of this study is not have high significant, but it give the new methed to predict mortality.

There are a few minor issues:
1. 1. The severity of hypertension and diabetes in the cohort was not expanded in stratified analyses.
Answer: According to your suggestion, we revised like below.

Second, data on the severity of hypertension and diabetes were not available. Instead, SBP and HbA1c were used as surrogates to adjust for blood pressure and diabetes severity.

2, BARD score is not well explained in the article.

Answer: According to your suggestion, we discuss BARD score like below.

. In this study, BARD score and APRI showed lower performance than NFS and FIB-4, but BARD score and APRI also have advantages. The BARD score can predict histological fibrosis with reasonable accuracy. Researchers reported that area under curve ROC of BARD score was 0.67 in predicting fibrosis, which was similar to 0.68 of NFS [6]. In addition, since APRI can be easily calculated with only two indicators (AST and platelets), the World Health Organization (WHO) recommends APRI to determine the stage of fibrosis in countries with limited resources [33]..

3, The impact of underlying diseases on mortality, such as the choice of therapeutic intervention and drug application, should be more addressed in the discussion.

Answer: According to your suggestion, we revised like below.

Finally, information on drugs for hypertension, diabetes, and hyperlipidemia has not been measured. Recently, some studies reported that the use of statin[40], angiotensin converting enzyme inhibitor (ACEI)[41], and diabetic medication[42] reduce the risk of advanced fibrosis.
